# Glucagon-like peptide-1 and peptide YY multi-agonism with GEP44 to optimize weight loss and glycemic control while reducing gastrointestinal side effects: the future of anti-obesity pharmacotherapy?

**DOI:** 10.3389/fendo.2026.1925534

**Published:** 2026-07-31

**Authors:** Melissa A. Burmeister, Skyler A. Brown, Jakeem J. Greer, Tyler J. Hoggatt, Whitney T. Zumwalt

**Affiliations:** 1 Department of Pharmaceutical Sciences, William Carey University School of Pharmacy, Biloxi, MS, United States; 2 Department of Pharmacy Practice, William Carey University School of Pharmacy, Biloxi, MS, United States

**Keywords:** anti-obesity medications, energy balance, gastrointestinal tolerability, GEP44, glucagon-like peptide-1 (GLP-1) receptor (GLP-1R) agonists (GLP-1RA), glucoregulation, monomeric chimeric multi-agonist, peptide YY (PYY)

## Abstract

Obesity is a chronic, multifactorial disease that has reached epidemic status. The glucagon-like peptide-1 (GLP-1) receptor (GLP-1R) is a key therapeutic target to achieve weight loss and glycemic control in obesity and type 2 diabetes mellitus. Tirzepatide and retatrutide, the latest generation of GLP-1-incorporating multi-agonists, target multiple receptors in complementary systems to achieve marked and sustained weight loss. However, the associated gastrointestinal (GI) side effects (*e.g.*, nausea, vomiting, diarrhea, abdominal pain) that often hinder patient compliance underscore a remaining need for therapies with better tolerability. GEP44, a novel, monomeric, chimeric peptide structurally based on native GLP-1, the GLP-1RA exendin-4 (Ex-4), and the gut-derived hormone peptide YY3-36 (PYY_3-36_), is an agonist at the GLP-1R and multiple neuropeptide Y (NPY) receptors. This review summarizes the design rationale for GEP44 and preclinical evidence supporting its beneficial metabolic effects and tolerability. Like GLP-1, PYY_3–36_ is associated with decreased food intake (FI), and co-administration of GLP-1RA and PYY_3–36_ elicits synergistic food intake (FI)- and body weight (BW)-reducing effects with similar or enhanced blood glucose (BG)-lowering effects. Studies examining the efficacy of GEP44 to promote weight loss and improve glucoregulation in lean vs. diet-induced obese rodents report that peripheral administration generally decreases BW, FI, and insulin resistance and increases EE at equivalent or greater magnitudes than those observed with single GLP-1RAs in a GLP-1R-dependent manner with improved GI tolerability. These beneficial actions reflect GEP44’s potential to expand the mechanistic scope of anti-obesity medications.

## Introduction

Obesity is a chronic, progressive, and relapsing disease of epidemic proportions. Numerous genetic, environmental, dietary, psychological, and psychosocial risk factors contribute to the development of obesity. Common obesity-related complications include type 2 diabetes mellitus (T2DM), cardiovascular disease, obstructive sleep apnea, osteoarthritis, certain cancers, and depression. Body weight (BW) regulation involves factors that control energy intake (FI) and energy expenditure (EE), with obesity developing when FI (*e.g.*, calories consumed) increases without a concomitant increase in EE (*e.g.*, physical activity, metabolism). Based on its anorexigenic and anti-hyperglycemic actions, the endogenous glucagon-like peptide-1 (GLP-1) system has emerged as key therapeutic target to achieve weight loss and enhance glycemic control in obesity and T2DM. The utility of endogenous GLP-1 is limited by its short half-life of only 1.5–3 minutes, owing to rapid degradation by the enzyme dipeptidyl peptidase 4 (DPP-IV) and renal clearance. This constraint of native GLP-1 was overcome by the development of DPP-IV-resistant GLP-1 analogs with extended half-lives, which display significant beneficial BW-and blood glucose (BG)- lowering effects. The GLP-1R agonist (GLP-1RA) exenatide (half-life=2.4 hours), prescribed for managing T2DM, also produced a modest loss of BW of generally 5% in individuals with or without T2DM (1). Subsequent long-acting monotherapies prescribed both as antihyperglycemic and anti-obesity medications (AOMs), such as liraglutide (half-life=13 hours) and semaglutide (half-life=165–184 hours), further reduce BW long-term, generally by 10% and 15%, respectively (2–5). However, these agents are associated with mild to moderate gastrointestinal (GI) side effects, including nausea, vomiting, diarrhea, and abdominal pain, which could hinder patient compliance and lead to discontinuation of treatment. A key challenge with discontinuation is rapid weight regain. Another obstacle to weight maintenance involves weight loss-induced activation of counterregulatory orexigenic mechanisms in the brain that promote hunger and energy conservation, which, in turn, reduce EE, prevent further weight loss, and promote weight gain. Although GLP-1RA treatment in patients reduces lean body mass (LBM), relative LBM remains the same, with a relative preservation of muscle strength (6, 7).

Further optimization of GLP-1RAs has involved targeting multiple receptors in complementary neurocircuits to achieve more marked and sustained weight loss while mitigating side effects to improve tolerance and compliance. Combination therapy (*i.e.*, co-administration of different compounds) or treatment with monomeric chimeric multi-agonists (*i.e.*, single molecules incorporating structural elements of two or three different incretin hormones) allows individual agents to be administered at low or subthreshold doses to achieve a synergistic therapeutic effect while circumventing the development of GI side effects. Furthermore, chimeric peptides exhibit improved pharmacokinetics and bioavailability and are, thus, more pharmacologically favorable than conjugated full-length peptides. Combination therapies are more effective than monotherapy at evoking sustained weight loss, partly due to their ability to suppress FI and prevent counter-regulatory orexigenic mechanisms. Tirzepatide, which targets both GLP-1R and glucose-dependent insulinotropic polypeptide (GIP) receptor (GIPR), causes a profound weight loss of ~20% (8). Retatrutide, which targets GLP-1R, GIPR, and glucagon receptor (GCGR), causes an overall weight loss of up to 25% (9). However, potentially problematic GI side effects persist with chimeric agents, underscoring a remaining need for more tolerable compounds (10).

GEP44 is a novel, monomeric, chimeric peptide structurally based on native GLP-1, the GLP-1RA exendin-4 (Ex-4), and the gut-derived hormone YY3-36 (PYY_3-36_). GEP44 targets multiple receptors in these complementary systems to regulate energy balance (EB) with fewer to no side effects as assessed in rodents (11). Specifically, it displays agonism at the GLP-1R and multiple neuropeptide Y (NPY) receptors (NPYRs) (11). The design rationale stems from preclinical and clinical observations that (1) PYY_3–36_ is associated with decreased FI and (2) co-administration of a GLP-1RA and PYY_3–36_ elicits synergistic FI- and BW-reducing (including reduced preference for high-fat diet) effects with either similar or enhanced glucose-lowering effects compared to administration of a GL1-RA or PYY_3–36_ alone (12–16). Of note, supraphysiological intravenous doses of PYY_3–36_ increase nausea with no enhanced anorectic effect in man (17). Here, the complementary actions of endogenous GLP-1 and PYY_3–36_ on EB and glucoregulation are reviewed. The design approach for constructing GEP44 is described, including validation assays. The efficacy of multi-agonism at GLP-1R and NPYRs by GEP44 (vs. GLP-1RA monotherapy) at improving weight loss, glucoregulation, and GI tolerability in preclinical studies, including the extent to which GLP-1R-mediated signaling is required, are addressed.

## Complementary beneficial metabolic actions of GLP-1 and PYY_3–36_ and dysregulation in obesity

GLP-1 and PYY_1–36_ are co-released from enteroendocrine L-cells of the distal colon postprandially (18–20). Endogenous GLP-1 is a 30- or 31-amino acid peptide derived from proglucagon, primarily circulating in active forms GLP-1 (7-36) amide or GLP-1 (7-37). GLP-1 is an incretin hormone that potently stimulates glucose-dependent insulin secretion. This insulinotropic effect is mediated by GLP-1R expressed on pancreatic β-cells (21). GLP-1 also inhibits glucagon secretion by pancreatic α-cells as well as promotes pancreatic β-cell proliferation and prevents apoptosis. GLP-1 promotes satiety and suppresses appetite by delaying gastric emptying and suppressing FI via peripheral and central GLP-1R (22). Specifically, anorexigenic effects are mediated by GLP-1R expressed on vagal afferent neurons (primarily nodose ganglia) and in brain regions that regulate energy homeostasis, including the arcuate nucleus (ARC) of the hypothalamus (22). The current consensus is that single and combination GLP-1RA-based therapies do not impact EE independent of weight loss (23). The physiologic actions of GLP-1 are mediated by intracellular G-protein-coupled signaling (Gα_s_) that leads to stimulation of adenylate cyclase, increased cytosolic cyclic AMP (cAMP), and activation of protein kinase A (24).

PYY is structurally related to NPY, an orexigenic peptide abundantly expressed in the hypothalamus (25). Full-length NPY_1-36_ and PYY_1-36_ (half-life=~9 mins) bind to NPYRs Y1R, Y2R, Y4R, and Y5R (25–27). Central NPY signaling largely regulates FI, while peripheral PYY signaling largely regulates glucoregulation (28, 29). NPY_1–36_ increases FI via hypothalamic Y5R, while PYY_1–36_ reduces insulin secretion rate and promotes β-cell survival via pancreatic Y1R (29). NPY_1–36_ and PYY_1–36_ are rapidly proteolyzed to NPY_3–36_ and PYY_3-36_ (primary active and circulating form; half-life = ~8–10 mins) cleavage products, respectively, by DPP-IV in the blood (30). NPY_3–36_ and PYY_3–36_ display high affinity for hypothalamic Y2R to inhibit FI (31). Of note, Y2R agonism within the ARC is more likely to be driven by NPY_3–36_ than PYY_3-36_ (32). The central anorexigenic effect of NPY_3–36_ and PYY_3–36_ involves inhibition of orexigenic NPY/agouti-related peptide neurons and associated disinhibition of anorexigenic proopiomelanocortin neurons (32–35). PYY_3–36_ acts at nodose ganglia Y2R to slow gastric motility (36). Additional signaling of PYY_3–36_ at peripheral Y2R increases insulin sensitivity by reducing lipotoxicity (via inhibition of hormone-sensitive lipase), promoting muscle glucose uptake (via GLUT4 translocation), and suppressing hepatic glucose production, both directly in fat and muscle as well as indirectly through brain-mediated mechanisms (29). Y2R receptor activation by PYY_3–36_ in L-cells also stimulates GLP-1 secretion, reinforcing its actions to improve glucose tolerance (37). The expression of Y2R throughout the intestine, on vagal afferents, and within the enteric nervous system suggests that the effects of PYY_3–36_ on active GLP-1 may also involve indirect hormonal or neural effects (38). Whereas mounting evidence indicates that PYY_3–36_ increases EE, PYY_1–36_ appears to have no such effect (39). The physiologic actions of PYY_3–36_ at Y2R are mediated by G_i/o_-coupled signaling that leads to decreased intracellular cAMP levels (29).

Reduced GLP-1 and PYY_3–36_ signaling play a role in the pathogenesis of obesity, with restorations to optimal circulating levels partly mediating the metabolic benefits of bariatric surgery. Postprandial GLP-1 secretion is impaired in individuals with obesity, and circulating levels rise following bariatric surgery (40). Both one-anastomosis gastric bypass and Roux-en-Y gastric bypass (RYGB) are associated with high postprandial secretions of gut hormones, particularly GLP-1, two years post-surgery (41). Circulating levels of PYY_3–36_ are commonly reduced in individuals with obesity and return to (or may exceed) levels seen in average-weight individuals following bariatric surgery, with increased PYY signaling mediating post-operative resolution of impaired glucose-mediated insulin and glucagon secretion (42–44). Of note, the metabolic benefits of RYGB are not attenuated in whole-body GLP-1R, Y2R, or combined GLP-1R/Y2R knockout diet-induced obese (DIO) mice, suggesting that alternate signaling pathways are also involved (45–47).

## Development of GEP44 and validation assays confirming receptor binding and functional activity

Preliminary studies tested six chimeric peptides, wherein the (1) N-terminal region amino acid residues were entirely (EP38 and EP45) or largely (EP40, EP44, EP46, and EP50) comprised of the N-terminal region amino acid residues present in Ex-4 and (2) C-terminal region amino acid residues were entirely comprised of the C-terminal region amino acid residues present in PYY_3-36_ (11). Additional modifications of G2S and E3Q were made in EP40, EP44, EP46, and EP50 to impart agonism at the glucagon receptor (GCGR) and, theoretically, achieve superior weight loss and metabolic improvements via increased EE (11). Initial screening entailed circular dichroism to determine structural integrity (*i.e.*, secondary structure, helicity, folding) (11). Further optimization led to the development of GEP44, wherein Ser2 was the D-isomer to prevent enzymatic degradation and four N-terminal region amino acid residues present in Ex-4 were substituted for those present in GLP-1 (Q13Y, M14L, L21E, E24A) to improve folding and receptor binding (11). Notably, the C-terminal region of the NPYR, which is critical for receptor binding, is highly conserved across the NPYR family (48). While GEP44 is designed to be Y1R- and Y2R-specific, its affinity at Y4 and Y5 remains unknown.

GEP44 potency and efficacy were examined in human embryonic kidney 293 cells expressing rat or human GLP-1R, human Y1R, human Y2R, or rat GCGR (11, 49). GLP-1R and GCGR agonist action was measured as the real-time increase in cytosolic cAMP concentration (11, 49). In contrast, Y1R and Y2R agonist action was measured as the decrease in cytosolic cAMP, with cells co-expressing endogenous adenosine A_2B_ receptors and recombinant Y1R and Y2R and initially treated with adenosine to raise cAMP levels (11, 49). These assays revealed that GEP44 binds to and activates GLP-1R, Y1R, and Y2R but not GCGR (11, 49). Receptor-selective agonism was confirmed by observations that GEP44 agonism was blocked by treatment with the GLP-1R antagonist Exendin-9–39 and Y2R antagonist BIIE0246 in cells expressing each receptor individually (11, 49).

Regarding biased agonism (*i.e.*, ligand-mediated activation of a subset of intracellular signaling pathways linked to a single receptor), GEP44 did not promote Y2R internalization (an indicator of receptor activation) (49). However, compared to Ex-4, GEP44 displayed similar efficacy regarding GLP-1R internalization and higher efficacy regarding β-arrestin recruitment (49). Of note, Ex-4 analogs with reduced capacity to elicit receptor internalization and β-arrestin recruitment are more efficacious at inducing insulin release than Ex-4, suggesting that ligand-induced insulin secretion tracks inversely with β-arrestin recruitment (50). Competitive binding of the peptides at GLP-1R against GLP-1 was also measured to gauge the effects of increased PYY components on GLP-1R binding. Compared to the Ex-4 reference competitor, GEP44 displayed moderate binding at GLP-1R despite also having comparable agonism at Y2R (11).

In isolated rat pancreatic islets treated with 10 mM glucose, glucose-stimulated insulin secretion was further increased by GEP44 (but not PYY_3-36_), although the GEP44-mediated enhancement of insulin secretion rate (ISR) was lower than that observed with Ex-4 (11). However, co-treatment with GEP44 and a Y1R antagonist elevated ISR closer to that observed with Ex-4 (49). In contrast to Ex-4, GEP44 did not stimulate glucose-induced cAMP production, nor did it inhibit glucagon secretion; however, it stimulated muscle glucose uptake (49). Similar to other GLP-1RAs, GEP44 increased glucose consumption and glycolytic flux (*i.e.*, rate of glycolysis) as reflected by lactate release (49). Microsomal stability assays in pooled rat liver microsomes determined that GEP44 and Ex-4 have similar metabolic stability to liver metabolism (mainly cytochrome P450) (11). Fluorescent *in-situ* hybridization and immunohistochemistry assays determined that IP- and ICV-delivered GEP44 colocalized with GLP-1R and Y1R in the area postrema (AP) and nucleus tractus solitarius (NTS), hindbrain regions involved in FI and control of nausea/malaise (49). Expression of GEP44 in cells expressing Y1R or Y2R was generally limited to the medial NTS, while GEP44 localization in cells expressing GLP-1R was generally restricted to the AP (49).

## Effects of GEP44 on energy balance and glucoregulation *in vivo*

The effects of GEP44 on FI (grams or kilocalories), EE, and glucose homeostasis as reported in five preclinical studies are summarized in **Table 1**. Initial dose escalation studies compared the effects of GEP44 on FI, BW, and glycemic control in lean and DIO rats fed standard chow or high-fat diet (60% kilocalories from fat), respectively, *ad libitum* (11, 49). In male lean rats, peripheral administration of GEP44 elicited a more robust reduction in FI than Ex-4 (11). Importantly, GEP44 was associated with only minimal GI side effects in this rodent study (11). Reduced nausea/malaise may be attributable to GEP44’s partial agonism at GLP-1R, which limits the intensity of emetic signaling in GLP-1R-expressing AP neurons (51). Furthermore, co-activation of Y2R-containing NTS neurons suppresses appetite and gastric emptying, promoting satiety (13). This balanced signaling in brainstem neuronal circuits mitigates the GI side effects observed with GLP-1R activation alone. In male DIO rats, peripheral administration of GEP44 resulted in sustained weight loss as well as reduced FI and fasting BG (FBG) (11, 49). GEP44 reduced glucose excursion and increased glucose clearance during an intraperitoneal glucose tolerance test at magnitudes greater than those observed with Ex-4 (11). In subsequent studies performed in male and female DIO rats, peripheral administration of GEP44 elicited more robust reductions in BW and FI than liraglutide and was also associated with decreased insulin resistance (52). Pair-feeding determined that the reduced BW, improved glucose tolerance, and decreased insulin resistance observed with GEP44 treatment were due directly to GEP44 signaling rather than a consequence of reduced FI and BW (52). Of note, pair-feeding reduced glucose tolerance in GEP44-treated animals (52).

**Table 1:** Preclinical studies examining the effects of GEP44 on body weight, food intake, energy expenditure, glucoregulation, and GI symptomatology compared to GLP-1RA monotherapy.

Publication year	Authors	Animal model(s)	Treatments (daily doses tested)	Parameters assessed (methodology)^†^	Main findings^‡^
2025	Goldberg et al. (57)	Male and female DIO rats	GEP44 (50 nmol/kg, s.c.) or Ex-4 (10 nmol/kg, s.c.) vs. Vehicle	BW, after 2-day treatment period FI, 2-day AVG EE, 2-day AVG (6-hr and 24-hr CT via telemetry; EE and RER via indirect calorimetry) Dark cycle vs. Light cycle AA, 2-day AVG (measured in conjunction with indirect calorimetry)	Male GEP44: ↓ BW by 3.8%, ↓ FI by 75%, ↓ 6-hr and 24-hr CT, ↓ Dark cycle AA, ↓ Light cycle AA, ↓ Dark cycle EE, ↑ Light cycle EE, ↓ Dark cycle RER, ↓ Light cycle RER Ex-4: ↓ BW by 3.4%, ↓ FI by 70%, ↓ 6-hr and 24-hr CT, ↓ Dark cycle AA, ↓ Light cycle AA, ↓ Dark cycle EE, ↑ Light cycle EE, ↓ Dark cycle RER, ↓ Light cycle RER Female GEP44: ↓ BW by 2.3%, ↓ FI by 65%, ↓ 6-hr and 24-hr CT, ↓ Dark cycle AA, ↔ Light cycle AA, ↓ Dark cycle EE, ↑ Light cycle EE, ↓ Dark cycle RER, ↓ Light cycle RER Ex-4: ↓ BW by 2.2%, ↓ FI by 70%, ↓ 6-hr and 12-hr CT, ↓ Dark cycle AA, ↔ Light cycle AA, ↓ Dark cycle EE, ↑ Light cycle EE, ↓ Dark cycle RER, ↓ Light cycle RER
2024	Blevins et al. (53)	Male and female DIO GLP-1R^+/+^ (WT) and GLP-1R^-/-^ (KO) mice	GEP44 (5–50 nmol/kg, s.c.) or Ex-4 (5–50 nmol/kg, s.c.) vs. Vehicle (unless otherwise specified)	BW after 3-day treatment period (vs. Pre-treatment baseline) FI, 3-day AVG EE, 3-day AVG (6-hr CT via telemetry) AA, 3-day AVG (gross motor activity in home cage) FBG, 2 hrs Post-treatment	Male WT GEP44 (50 nmol/kg): ↓ BW by 4.3%, ↓ FI by 43%, ↓ FBG by 30%, ↓ CT, ↓ AA Ex-4 (50 nmol/kg): ↓ BW by 3.4%, ↓ FI by 36%, ↓ FBG by 40%, ↔ CT, ↓ AA Male KO GEP44 (50 nmol/kg): ↔ BW, ↓ FI by 15%, ↔ FBG, ↓ CT, ↓ AA Ex-4 (50 nmol/kg): ↔ BW, ↔ FI, ↔ FBG, ↔ CT, ↔ AA Female WT GEP44 (50 nmol/kg): ↓ BW by 4%, ↓ FI by 36%, ↓ FBG by 35%, ↓ CT, ↓ AA Ex-4 (50 nmol/kg): ↓ BW by 4.7%, ↓ FI by 36%, ↓ FBG by 50%, ↓ CT, ↓ AA Female KO GEP44 (50 nmol/kg): ↔ BW, ↔ FI, ↔ FBG, ↔ CT, ↔ AA Ex-4 (50 nmol/kg): ↔ BW, ↔ FI, ↔ FBG, ↔ CT, ↔ AA
2024	Elfers et al. (52)	Male and female DIO rats	GEP44 or Liraglutide (5-50 nmol/kg, s.c.) vs. Vehicle (unless otherwise specified)	BW, after 28-day treatment period FI, AVG over 28-day treatment period HOMA-IR, after 28-day treatment period (vs. Pre-treatment baseline)	Male GEP44 (50 nmol/kg): ↓ BW by 16%; ↓ FI by 38.5%; ↓ HOMA-IR by 60% Liraglutide (50 nmol/kg): ↓ BW by 10%; ↓ FI by 19%; ↓ HOMA-IR by 58% Female GEP44 (50 nmol/kg): ↓ BW by 12%; ↓ FI by 36%; ↔ HOMA-IR Liraglutide (50 nmol/kg): ↓ BW by 6%; ↓ FI by 18%; ↔ HOMA-IR
2023	Chichura et al. (49)	Male DIO rats	GEP44 (0.5–100 nmol/kg, s.c.) or Ex-4 (0.5–20 nmol/kg, s.c.) vs. Vehicle (unless otherwise specified)	FI, AVG over 3-day treatment period (vs. Pre-treatment baseline) FI, 24-hr AVG over 3-day treatment period	GEP44 (20 nmol/kg): ↓ FI by 75% Ex-4 (20 nmol/kg): ↓ FI by 55% GEP44 (5 nmol/kg): ↓ FI by 55%; (10 nmol/kg): ↓ FI by 75%
2021	Milliken et al. (11)	Male lean rats	GEP44 (0.3–60 nmol/kg, s.c.) or Ex-4 (0.6–60 nmol/kg,s.c.) vs. Vehicle	FI, 2-day AVG KI, 2-day AVG	GEP44 (60 nmol/kg): ↓ FI by 90%; KI: 1 g Ex-4 (60 nmol/kg): ↓ FI by 52.5%; KI: 9 g
		Male DIO rats	GEP44 (10 nmol/kg, s.c.) or Ex-4 (10 nmol/kg, s.c.) or Vehicle vs. Pre-treatment baseline	BW, after 5-day treatment period FI, 24-hr AVG over 5-day treatment period FBG, after 5-day treatment period Glucose tolerance, after 5-day treatment period (IPGTT)	GEP44: ↓ BW by 5.5%; ↓ FI by 56%; ↓ FBG by 11%; ↓ BG AUC by 33% Ex-4: ↓ BW by 4%; ↓ FI by 42%; ↓ FBG by 12%; ↔ BG AUC
		Mammalian musk shrew	GEP44 (10 or 60 nmol/kg, i.p.) or Ex-4 (5 nmol/kg, i.p.) vs. Vehicle	Glucose tolerance (IPGTT) Emesis	GEP44 (10 nmol/kg): ↓ BG AUC by 27%; (10 or 60 nmol): 0 emetic episodes Ex-4: 3 emetic episodes

AA, ambulatory activity; AUC, area under curve; AVG, average; BG, blood glucose; BW, body weight; CT, core temperature; DIO, diet-induced obese; EE, energy expenditure; FBG, fasting blood glucose; Ex-4: exendin-4; FI, food intake; HOMA-IR, homeostatic model assessment for insulin resistance; KI, kaolin intake; KO, knockout; i.p., intraperitoneal; IPGTT, intraperitoneal glucose tolerance test; RER, respiratory exchange ratio; s.c.: subcutaneous; WT, wild-type. Vehicle = saline.

**^†^**Food was provided *ad libitum*. Indirect calorimetry was performed using a Comprehensive Lab Animal Monitoring System (CLAMS, Columbus Instruments, Columbus, OH, USA).

**^‡^**Approximate values for select doses presented. ↑: significantly increased, p < 0.05; ↓: significantly decreased, p < 0.05; ↔: no significant change, p > 0.05. Directional changes only provided for CT, EE, AA, and RER.

Follow-up studies examined EB as well as core temperature (CT), ambulatory activity (AA), and non-shivering thermogenesis to estimate changes in EE, along with FBG and plasma levels of glucagon, leptin, and total cholesterol as additional markers of metabolic status, in both GLP-1R wild-type (GLP-1R^+/+^) and whole-body knockout (GLP-1R^-/-^) mice (53). The robust GEP44- and Ex-4-mediated reductions in BW observed in male and female GLP-1R^+/+^ mice were absent in GLP-1R^-/-^ counterparts (53). GEP44 and Ex-4 treatment significantly reduced FI only in male and female GLP-1R^+/+^ mice (even when normalized to BW) (53). Of note, the lowest doses of GEP44 and Ex-4 tested (5 nmol/kg) and highest dose of GEP44 tested (50 nmol/kg) significantly reduced FI in male GLP-1R^-/-^ mice (53). Although these data indicate that GEP44’s effect on BW is primarily mediated by GLP-1R, co-activation of Y2R remains crucial given the anti-emetic actions and increased metabolic effectiveness at a lower dose (54). CT was generally lower with GEP44 and Ex-4 treatment in GLP-1R^+/+^ animals, which was likely due, at least in part, to reduced FI and associated loss of the thermic effect of food (53). Treatment-induced reductions in CT as well as in AA and FBG were largely absent in GLP-1R^-/-^ animals (53). However, intrascapular brown adipose tissue (IBAT) temperature, a measure of thermogenic activity, was stimulated in GEP44- and Ex-4-treated male GLP-1R^+/+^ DIO animals (53). These paradoxical events occur because activation of hypothalamic GLP-1R promotes non-shivering IBAT thermogenesis via increased sympathetic outflow; reduced FI and BW, in turn, lower CT to conserve energy (55, 56). Regarding changes in thermogenic gene expression in IBAT, peroxisome proliferator-activated receptor gamma coactivator one-alpha (*Ppargc1a*), a master regulator of energy metabolism, was upregulated in GEP44-treated male GLP-1R^+/+^ mice but unchanged in GLP-1R^-/-^ counterparts (53). *Ppargc1a* was also upregulated in GEP44-treated female GLP-1R^+/+^ mice, as were uncoupling protein 1 (*Ucp1*), G-protein-coupled receptor 120 (*Gpr120*), and *Ppargc1a* in Ex-4 treated animals; except for *Gpr120*, these changes were absent in GLP-1R^-/-^ counterparts (53). Despite the incongruencies observed in CT and IBAT, these findings indicate that GLP-1R activation by GEP44 or Ex-4 promotes thermogenesis overall.

Indirect calorimetry revealed that GEP44 and Ex-4 exerted time of day-dependent changes in EE in male and female DIO rats, wherein EE was reduced during the dark cycle yet increased during the light cycle (57). Regardless of treatment and sex, this effect on EE was coupled with marked reductions in BW, FI, CT, AA, and respiratory exchange ratio (RER), indicating increased utilization of fat for fuel rather than carbohydrates (57). Overall, brown adipose tissue (BAT) thermogenesis and EE were reduced with both GEP44 and Ex-4 treatment, which may have been secondary to reductions in diet-induced thermogenesis (57). These observations confirmed previous reports that GLP-1RA-elicted weight loss is primarily due to reduced FI and may reflect a key counterregulatory mechanism that limits the overall efficacy of GEP44 and Ex-4 to safeguard against excessive weight loss (57). Plasma levels of metabolic hormones were altered by GEP44 administration in a sex-specific manner (57). In males, plasma insulin and cholesterol were increased, whereas plasma glucose and adiponectin were decreased. In females, plasma insulin, fibroblast growth factor 21 (a hepatic hormone that increases EE), and free fatty acids were increased, whereas plasma glucose and adiponectin were decreased (57). Similar trends were observed with Ex-4 (57). The GEP44- and Ex-4 mediated reductions in plasma adiponectin levels in an animal model of obesity are in contrast to other rodent and clinical findings that GLP-1RAs either do not change or generally increase plasma adiponectin levels, which is associated with weight loss (including reduced fat mass) and improved metabolic health (including improved insulin sensitivity) (5, 58, 59).

## Conclusion

The most effective AOMs will likely target multiple pathways involved in regulating EB with favorable tolerability. PYY_3–36_ displays potential to be a safe and efficacious complementary pharmacotherapy to GLP-1RAs (14, 16). No clinical trials have assessed the actions of GEP44 on EB and glucoregulation in patients. In April 2026, it was announced that GEP44 would be entering Investigational New Drug (IND)-enabling pharmacology and toxicology studies per the U.S. Food and Drug Administration. The efficacy, safety, and tolerability of other agents targeting GLP-1R and/or NPY receptors long-term has been assessed (48, 60–64). In preclinical studies, treatment with a single peptide conjugate targeting GLP-1R and Y2R reduced FI and BW as well as increased insulin sensitivity by magnitudes considerably greater than those elicited by semaglutide and with less nausea (60). Sustained activation of Y1R or Y2R in mice imparts metabolic benefits (48, 61). However, treatment with the long-acting Y2R agonist BI 1820237 (development discontinued in 2024) was associated with transient nausea and vomiting at high doses in men with overweight or obesity, with no differences in tolerability observed when combined with liraglutide (62). Preclinically, NPY2R agonism with BI 1820237 did not affect BW, but synergistic NPY2R and GCGR/GLP-1R agonism with BI 1820237 and survodutide, respectively, reduced BW more than survodutide alone (63). Targeting both GLP-1R and Y4R is beneficial in mouse models of Alzheimer’s disease (64). These and other next-generation AOMs, thus, show promise to expand and refine the armamentarium of pharmacotherapeutic agents for reducing obesity-related morbidity and mortality.
